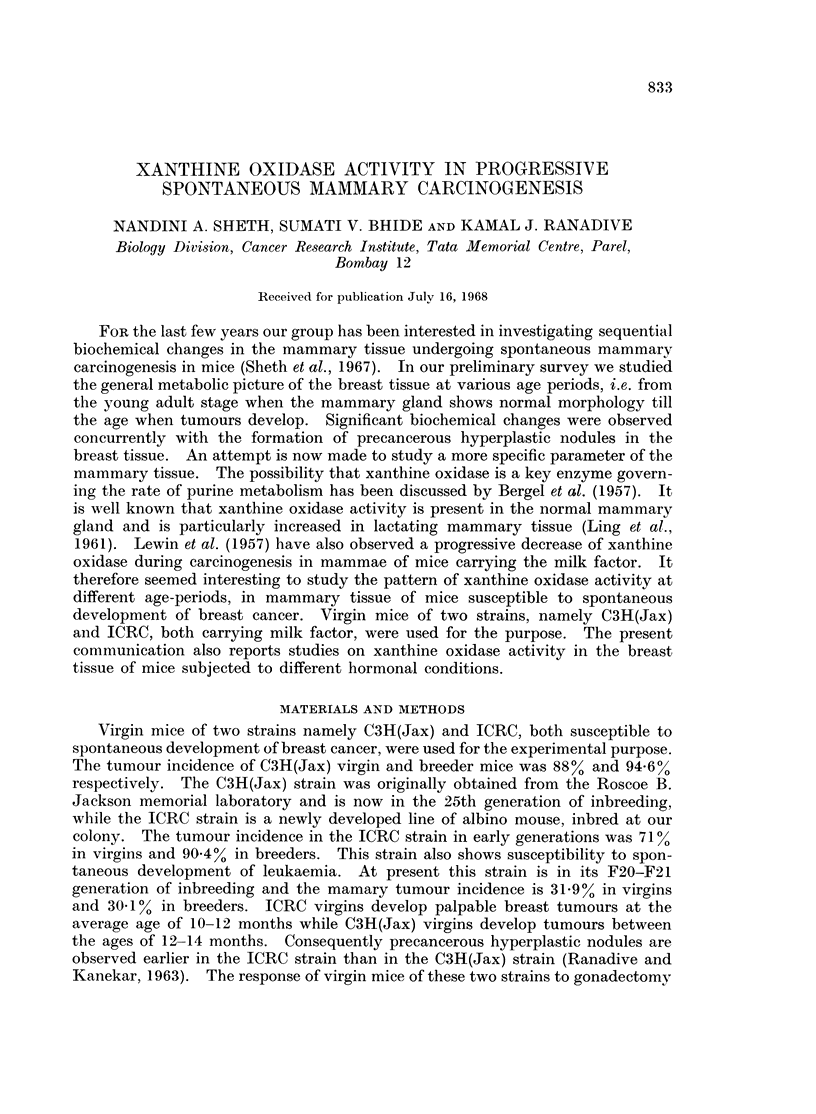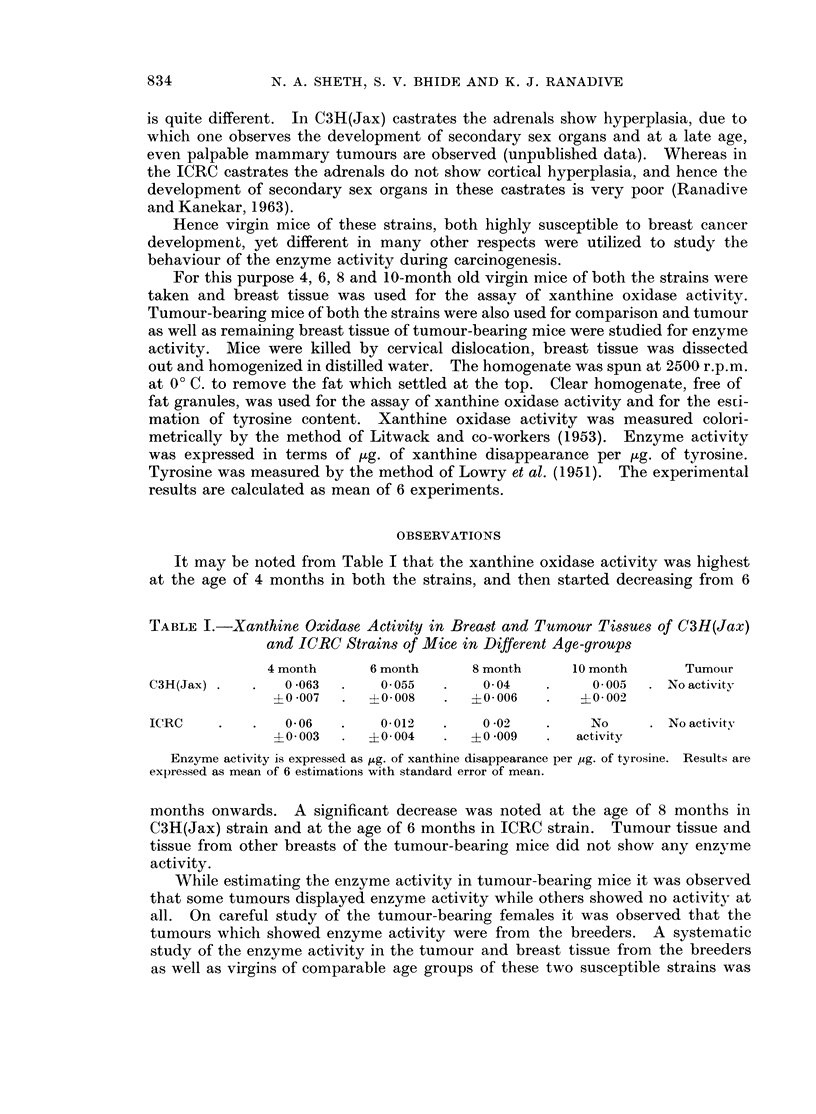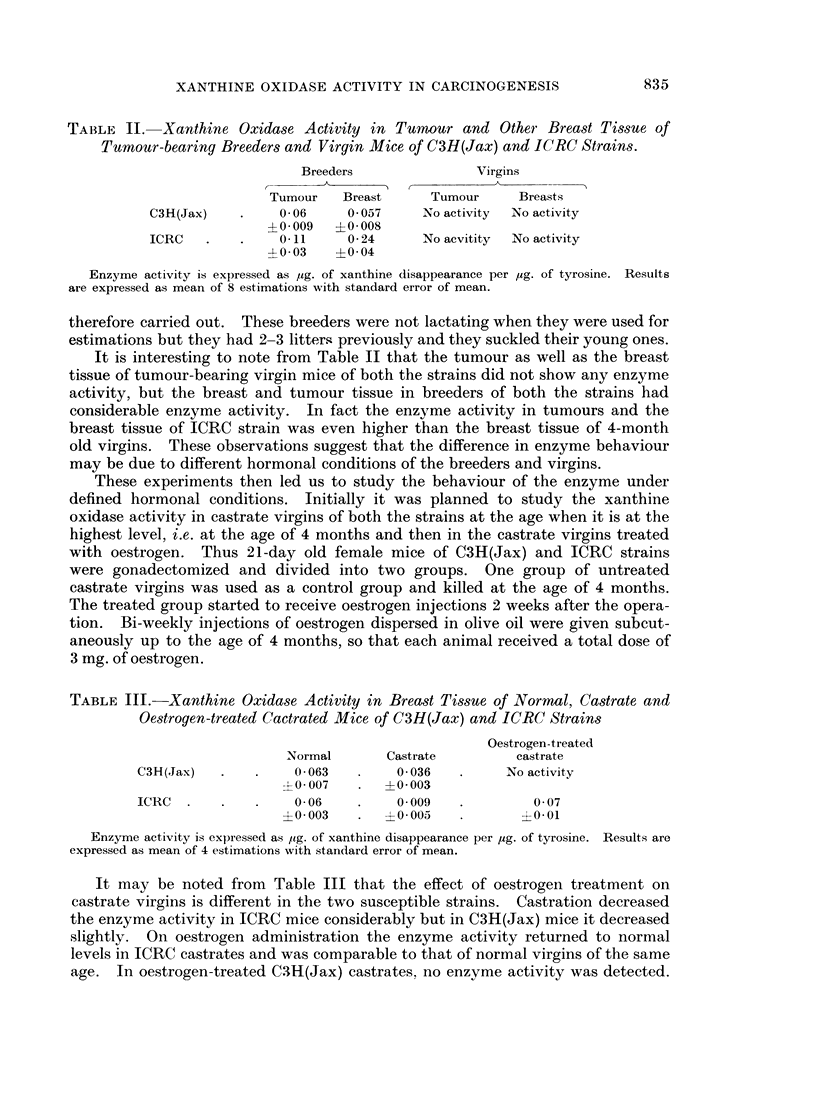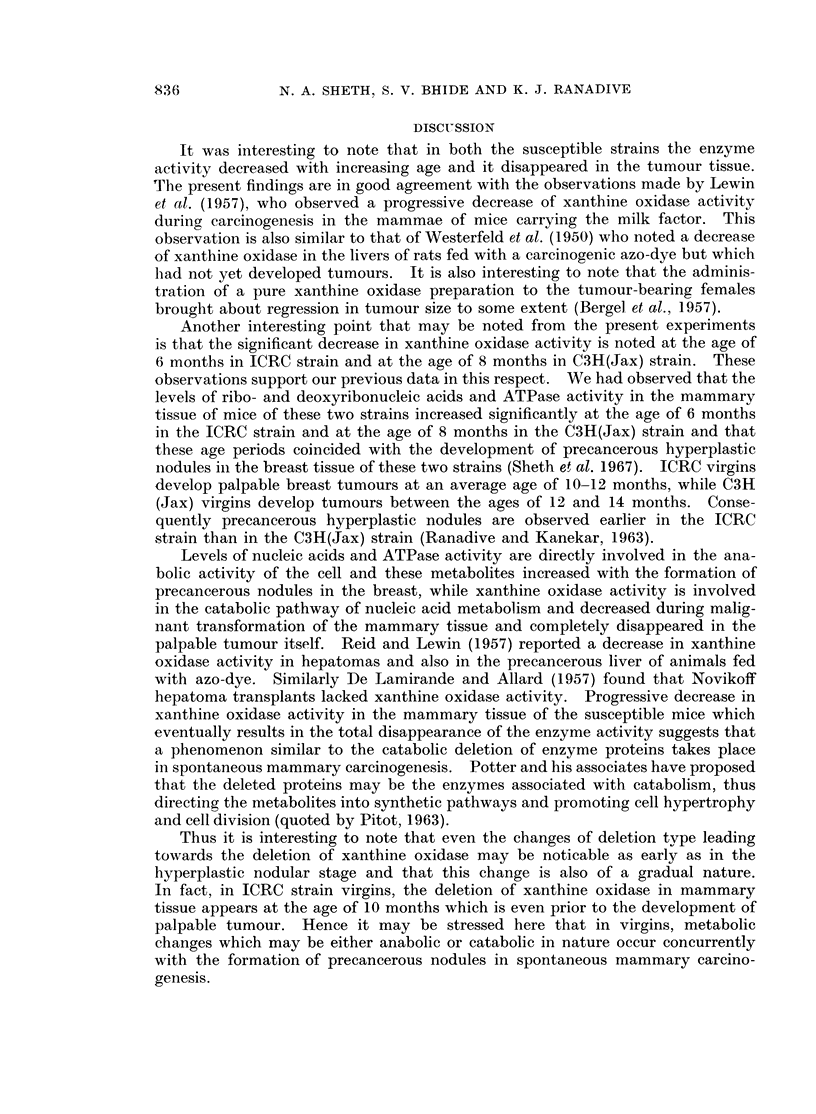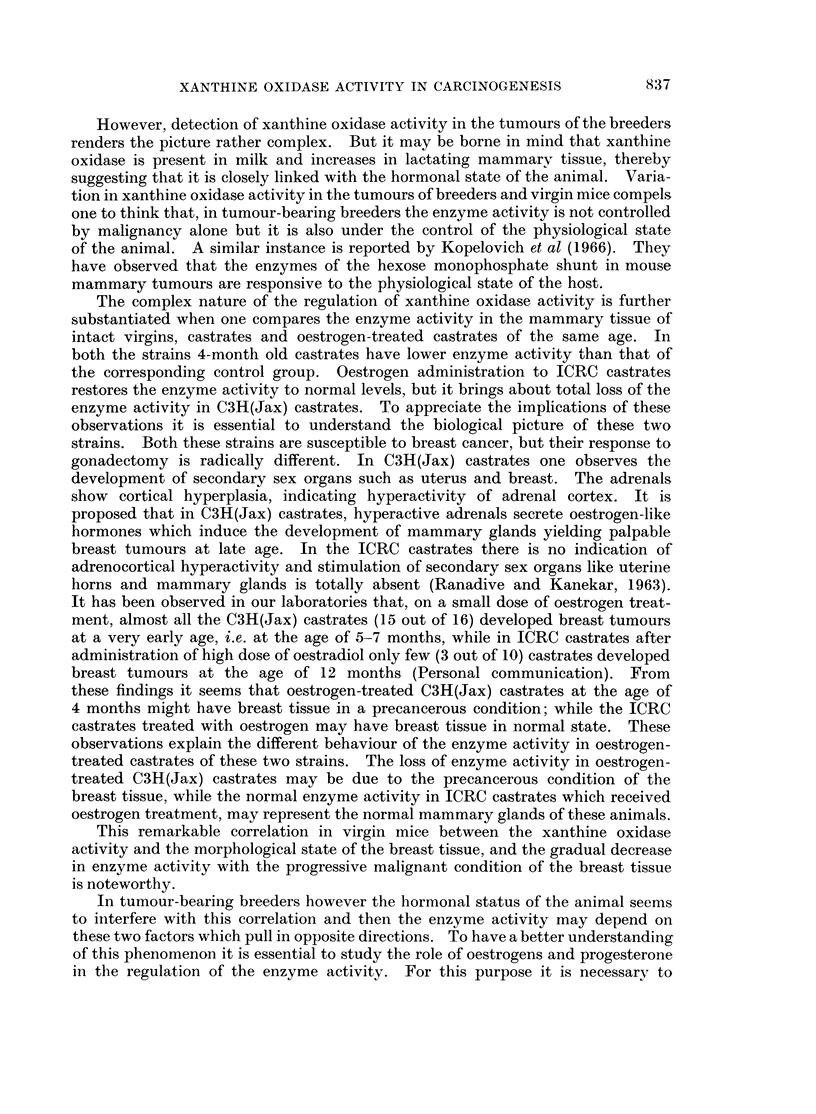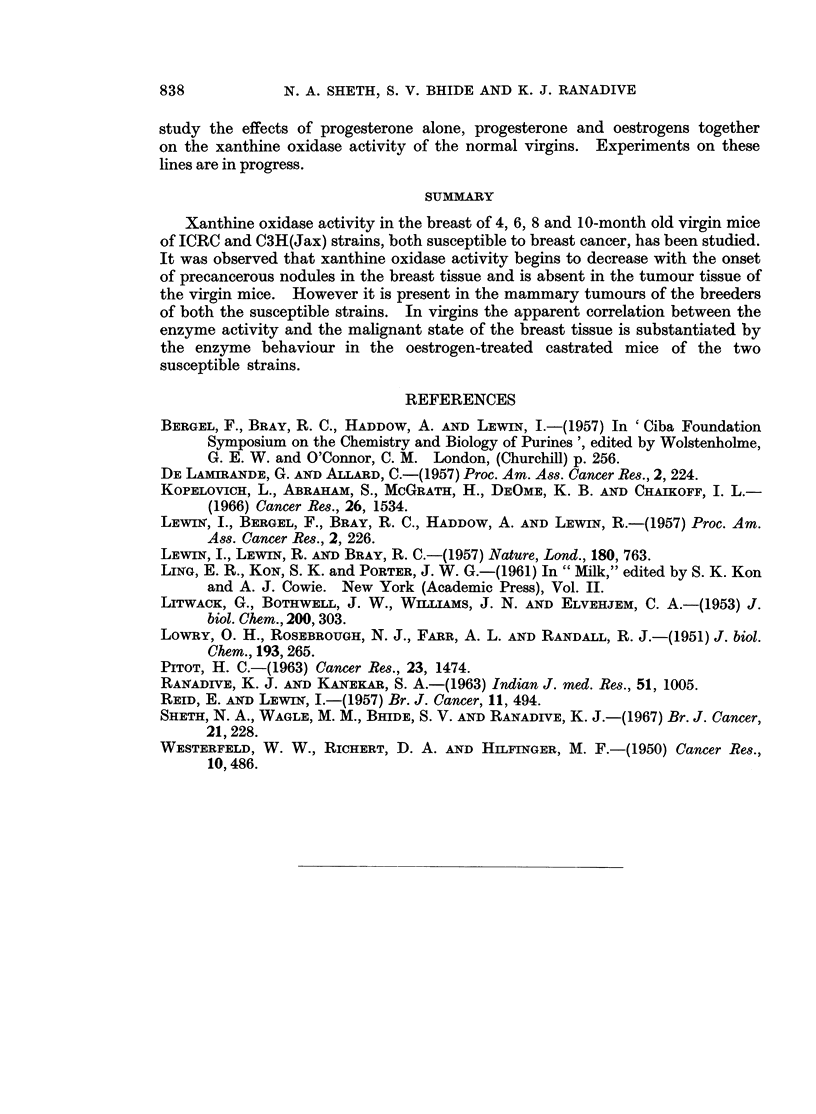# Xanthine oxidase acitivity in progressive spontaneous mammary carcinogenesis.

**DOI:** 10.1038/bjc.1968.97

**Published:** 1968-12

**Authors:** N. A. Sheth, S. V. Bhide, K. J. Ranadive


### XANTHINE OXIDASE ACTIVITY IN PROGRESSIVE SPONTANEOUS MAMMARY CARCINOGENESIS

NANDINI A. SHETH, SUMATI V. BHIDE AND KAMAL J. RANADIVE

*Biology Division, Cancer Research Institute, Tata Memorial Centre, Parel, Bombay* 12

Received for publication July 16, 1968

FOR the last few years our group has been interested in investigating sequential biochemical changes in the mammary tissue undergoing spontaneous mammary carcinogenesis in mice (Sheth *et al.*, 1967). In our preliminary survey we studied the general metabolic picture of the breast tissue at various age periods, *i.e.* from the young adult stage when the mammary gland shows normal morphology till the age when tumours develop. Significant biochemical changes were observed concurrently with the formation of precancerous hyperplastic nodules in the breast tissue. An attempt is now made to study a more specific parameter of the mammary tissue. The possibility that xanthine oxidase is a key enzyme governing the rate of purine metabolism has been discussed by Bergel *et al.* (1957). It is well known that xanthine oxidase activity is present in the normal mammary gland and is particularly increased in lactating mammary tissue (Ling *et al.*, 1961). Lewin *et al.* (1957) have also observed a progressive decrease of xanthine oxidase during carcinogenesis in mammae of mice carrying the milk factor. It therefore seemed interesting to study the pattern of xanthine oxidase activity at different age-periods, in mammary tissue of mice susceptible to spontaneous development of breast cancer. Virgin mice of two strains, namely C3H(Jax) and ICRC, both carrying milk factor, were used for the purpose. The present communication also reports studies on xanthine oxidase activity in the breast tissue of mice subjected to different hormonal conditions.

### MATERIALS AND METHODS

Virgin mice of two strains namely C3H(Jax) and ICRC, both susceptible to spontaneous development of breast cancer, were used for the experimental purpose. The tumour incidence of C3H(Jax) virgin and breeder mice was 88% and 94·6% respectively. The C3H(Jax) strain was originally obtained from the Roscoe B. Jackson memorial laboratory and is now in the 25th generation of inbreeding, while the ICRC strain is a newly developed line of albino mouse, inbred at our colony. The tumour incidence in the ICRC strain in early generations was 71% in virgins and 90·4% in breeders. This strain also shows susceptibility to spontaneous development of leukaemia. At present this strain is in its F20–F21 generation of inbreeding and the mamary tumour incidence is 31·9% in virgins and 30·1% in breeders. ICRC virgins develop palpable breast tumours at the average age of 10–12 months while C3H(Jax) virgins develop tumours between the ages of 12–14 months. Consequently precancerous hyperplastic nodules are observed earlier in the ICRC strain than in the C3H(Jax) strain (Ranadive and Kanekar, 1963). The response of virgin mice of these two strains to gonadectomy is quite different. In C3H(Jax) castrates the adrenals show hyperplasia, due to which one observes the development of secondary sex organs and at a late age, even palpable mammary tumours are observed (unpublished data). Whereas in the ICRC castrates the adrenals do not show cortical hyperplasia, and hence the development of secondary sex organs in these castrates is very poor (Ranadive and Kanekar, 1963).

Hence virgin mice of these strains, both highly susceptible to breast cancer development, yet different in many other respects were utilized to study the behaviour of the enzyme activity during carcinogenesis.

For this purpose 4, 6, 8 and 10-month old virgin mice of both the strains were taken and breast tissue was used for the assay of xanthine oxidase activity. Tumour-bearing mice of both the strains were also used for comparison and tumour as well as remaining breast tissue of tumour-bearing mice were studied for enzyme activity. Mice were killed by cervical dislocation, breast tissue was dissected out and homogenized in distilled water. The homogenate was spun at 2500 r.p.m. at 0° C. to remove the fat which settled at the top. Clear homogenate, free of fat granules, was used for the assay of xanthine oxidase activity and for the estimation of tyrosine content. Xanthine oxidase activity was measured colorimetrically by the method of Litwack and co-workers (1953). Enzyme activity was expressed in terms of $\mu$g. of xanthine disappearance per $\mu$g. of tyrosine. Tyrosine was measured by the method of Lowry *et al.* (1951). The experimental results are calculated as mean of 6 experiments.

### OBSERVATIONS

It may be noted from Table I that the xanthine oxidase activity was highest at the age of 4 months in both the strains, and then started decreasing from 6

TABLE I.—*Xanthine Oxidase Activity in Breast and Tumour Tissues of C3H(Jax) and ICRC Strains of Mice in Different Age-groups*

| | 4 month | 6 month | 8 month | 10 month | Tumour |
|---|---|---|---|---|---|
| C3H(Jax) | 0·063 ±0·007 | 0·055 ±0·008 | 0·04 ±0·006 | 0·005 ±0·002 | No activity |
| ICRC | 0·06 ±0·003 | 0·012 ±0·004 | 0·02 ±0·009 | No activity | No activity |

Enzyme activity is expressed as $\mu$g. of xanthine disappearance per $\mu$g. of tyrosine. Results are expressed as mean of 6 estimations with standard error of mean.

months onwards. A significant decrease was noted at the age of 8 months in C3H(Jax) strain and at the age of 6 months in ICRC strain. Tumour tissue and tissue from other breasts of the tumour-bearing mice did not show any enzyme activity.

While estimating the enzyme activity in tumour-bearing mice it was observed that some tumours displayed enzyme activity while others showed no activity at all. On careful study of the tumour-bearing females it was observed that the tumours which showed enzyme activity were from the breeders. A systematic study of the enzyme activity in the tumour and breast tissue from the breeders as well as virgins of comparable age groups of these two susceptible strains was

TABLE II.—*Xanthine Oxidase Activity in Tumour and Other Breast Tissue of Tumour-bearing Breeders and Virgin Mice of C3H(Jax) and ICRC Strains.*

| | Breeders | | Virgins | |
|---|---|---|---|---|
| | Tumour | Breast | Tumour | Breasts |
| C3H(Jax) . | 0·06 ±0·009 | 0·057 ±0·008 | No activity | No activity |
| ICRC . . | 0·11 ±0·03 | 0·24 ±0·04 | No acvitity | No activity |

Enzyme activity is expressed as μg. of xanthine disappearance per μg. of tyrosine. Results are expressed as mean of 8 estimations with standard error of mean.

therefore carried out. These breeders were not lactating when they were used for estimations but they had 2–3 litters previously and they suckled their young ones.

It is interesting to note from Table II that the tumour as well as the breast tissue of tumour-bearing virgin mice of both the strains did not show any enzyme activity, but the breast and tumour tissue in breeders of both the strains had considerable enzyme activity. In fact the enzyme activity in tumours and the breast tissue of ICRC strain was even higher than the breast tissue of 4-month old virgins. These observations suggest that the difference in enzyme behaviour may be due to different hormonal conditions of the breeders and virgins.

These experiments then led us to study the behaviour of the enzyme under defined hormonal conditions. Initially it was planned to study the xanthine oxidase activity in castrate virgins of both the strains at the age when it is at the highest level, *i.e.* at the age of 4 months and then in the castrate virgins treated with oestrogen. Thus 21-day old female mice of C3H(Jax) and ICRC strains were gonadectomized and divided into two groups. One group of untreated castrate virgins was used as a control group and killed at the age of 4 months. The treated group started to receive oestrogen injections 2 weeks after the operation. Bi-weekly injections of oestrogen dispersed in olive oil were given subcutaneously up to the age of 4 months, so that each animal received a total dose of 3 mg. of oestrogen.

TABLE III.—*Xanthine Oxidase Activity in Breast Tissue of Normal, Castrate and Oestrogen-treated Cactrated Mice of C3H(Jax) and ICRC Strains*

| | Normal | Castrate | Oestrogen-treated castrate |
|---|---|---|---|
| C3H(Jax) . . | 0·063 ±0·007 | 0·036 ±0·003 | No activity |
| ICRC . . . | 0·06 ±0·003 | 0·009 ±0·005 | 0·07 ±0·01 |

Enzyme activity is expressed as μg. of xanthine disappearance per μg. of tyrosine. Results are expressed as mean of 4 estimations with standard error of mean.

It may be noted from Table III that the effect of oestrogen treatment on castrate virgins is different in the two susceptible strains. Castration decreased the enzyme activity in ICRC mice considerably but in C3H(Jax) mice it decreased slightly. On oestrogen administration the enzyme activity returned to normal levels in ICRC castrates and was comparable to that of normal virgins of the same age. In oestrogen-treated C3H(Jax) castrates, no enzyme activity was detected.

DISCUSSION

It was interesting to note that in both the susceptible strains the enzyme activity decreased with increasing age and it disappeared in the tumour tissue. The present findings are in good agreement with the observations made by Lewin *et al.* (1957), who observed a progressive decrease of xanthine oxidase activity during carcinogenesis in the mammae of mice carrying the milk factor. This observation is also similar to that of Westerfeld *et al.* (1950) who noted a decrease of xanthine oxidase in the livers of rats fed with a carcinogenic azo-dye but which had not yet developed tumours. It is also interesting to note that the administration of a pure xanthine oxidase preparation to the tumour-bearing females brought about regression in tumour size to some extent (Bergel *et al.*, 1957).

Another interesting point that may be noted from the present experiments is that the significant decrease in xanthine oxidase activity is noted at the age of 6 months in ICRC strain and at the age of 8 months in C3H(Jax) strain. These observations support our previous data in this respect. We had observed that the levels of ribo- and deoxyribonucleic acids and ATPase activity in the mammary tissue of mice of these two strains increased significantly at the age of 6 months in the ICRC strain and at the age of 8 months in the C3H(Jax) strain and that these age periods coincided with the development of precancerous hyperplastic nodules in the breast tissue of these two strains (Sheth *et al.* 1967). ICRC virgins develop palpable breast tumours at an average age of 10–12 months, while C3H (Jax) virgins develop tumours between the ages of 12 and 14 months. Consequently precancerous hyperplastic nodules are observed earlier in the ICRC strain than in the C3H(Jax) strain (Ranadive and Kanekar, 1963).

Levels of nucleic acids and ATPase activity are directly involved in the anabolic activity of the cell and these metabolites increased with the formation of precancerous nodules in the breast, while xanthine oxidase activity is involved in the catabolic pathway of nucleic acid metabolism and decreased during malignant transformation of the mammary tissue and completely disappeared in the palpable tumour itself. Reid and Lewin (1957) reported a decrease in xanthine oxidase activity in hepatomas and also in the precancerous liver of animals fed with azo-dye. Similarly De Lamirande and Allard (1957) found that Novikoff hepatoma transplants lacked xanthine oxidase activity. Progressive decrease in xanthine oxidase activity in the mammary tissue of the susceptible mice which eventually results in the total disappearance of the enzyme activity suggests that a phenomenon similar to the catabolic deletion of enzyme proteins takes place in spontaneous mammary carcinogenesis. Potter and his associates have proposed that the deleted proteins may be the enzymes associated with catabolism, thus directing the metabolites into synthetic pathways and promoting cell hypertrophy and cell division (quoted by Pitot, 1963).

Thus it is interesting to note that even the changes of deletion type leading towards the deletion of xanthine oxidase may be noticable as early as in the hyperplastic nodular stage and that this change is also of a gradual nature. In fact, in ICRC strain virgins, the deletion of xanthine oxidase in mammary tissue appears at the age of 10 months which is even prior to the development of palpable tumour. Hence it may be stressed here that in virgins, metabolic changes which may be either anabolic or catabolic in nature occur concurrently with the formation of precancerous nodules in spontaneous mammary carcinogenesis.

However, detection of xanthine oxidase activity in the tumours of the breeders renders the picture rather complex. But it may be borne in mind that xanthine oxidase is present in milk and increases in lactating mammary tissue, thereby suggesting that it is closely linked with the hormonal state of the animal. Variation in xanthine oxidase activity in the tumours of breeders and virgin mice compels one to think that, in tumour-bearing breeders the enzyme activity is not controlled by malignancy alone but it is also under the control of the physiological state of the animal. A similar instance is reported by Kopelovich *et al* (1966). They have observed that the enzymes of the hexose monophosphate shunt in mouse mammary tumours are responsive to the physiological state of the host.

The complex nature of the regulation of xanthine oxidase activity is further substantiated when one compares the enzyme activity in the mammary tissue of intact virgins, castrates and oestrogen-treated castrates of the same age. In both the strains 4-month old castrates have lower enzyme activity than that of the corresponding control group. Oestrogen administration to ICRC castrates restores the enzyme activity to normal levels, but it brings about total loss of the enzyme activity in C3H(Jax) castrates. To appreciate the implications of these observations it is essential to understand the biological picture of these two strains. Both these strains are susceptible to breast cancer, but their response to gonadectomy is radically different. In C3H(Jax) castrates one observes the development of secondary sex organs such as uterus and breast. The adrenals show cortical hyperplasia, indicating hyperactivity of adrenal cortex. It is proposed that in C3H(Jax) castrates, hyperactive adrenals secrete oestrogen-like hormones which induce the development of mammary glands yielding palpable breast tumours at late age. In the ICRC castrates there is no indication of adrenocortical hyperactivity and stimulation of secondary sex organs like uterine horns and mammary glands is totally absent (Ranadive and Kanekar, 1963). It has been observed in our laboratories that, on a small dose of oestrogen treatment, almost all the C3H(Jax) castrates (15 out of 16) developed breast tumours at a very early age, *i.e.* at the age of 5–7 months, while in ICRC castrates after administration of high dose of oestradiol only few (3 out of 10) castrates developed breast tumours at the age of 12 months (Personal communication). From these findings it seems that oestrogen-treated C3H(Jax) castrates at the age of 4 months might have breast tissue in a precancerous condition; while the ICRC castrates treated with oestrogen may have breast tissue in normal state. These observations explain the different behaviour of the enzyme activity in oestrogen-treated castrates of these two strains. The loss of enzyme activity in oestrogen-treated C3H(Jax) castrates may be due to the precancerous condition of the breast tissue, while the normal enzyme activity in ICRC castrates which received oestrogen treatment, may represent the normal mammary glands of these animals.

This remarkable correlation in virgin mice between the xanthine oxidase activity and the morphological state of the breast tissue, and the gradual decrease in enzyme activity with the progressive malignant condition of the breast tissue is noteworthy.

In tumour-bearing breeders however the hormonal status of the animal seems to interfere with this correlation and then the enzyme activity may depend on these two factors which pull in opposite directions. To have a better understanding of this phenomenon it is essential to study the role of oestrogens and progesterone in the regulation of the enzyme activity. For this purpose it is necessary to study the effects of progesterone alone, progesterone and oestrogens together on the xanthine oxidase activity of the normal virgins. Experiments on these lines are in progress.

### SUMMARY

Xanthine oxidase activity in the breast of 4, 6, 8 and 10-month old virgin mice of ICRC and C3H(Jax) strains, both susceptible to breast cancer, has been studied. It was observed that xanthine oxidase activity begins to decrease with the onset of precancerous nodules in the breast tissue and is absent in the tumour tissue of the virgin mice. However it is present in the mammary tumours of the breeders of both the susceptible strains. In virgins the apparent correlation between the enzyme activity and the malignant state of the breast tissue is substantiated by the enzyme behaviour in the oestrogen-treated castrated mice of the two susceptible strains.

### REFERENCES

Bergel, F., Bray, R. C., Haddow, A. and Lewin, I.—(1957) In ' Ciba Foundation Symposium on the Chemistry and Biology of Purines ', edited by Wolstenholme, G. E. W. and O'Connor, C. M. London, (Churchill) p. 256.

De Lamirande, G. and Allard, C.—(1957) *Proc. Am. Ass. Cancer Res.*, **2**, 224.

Kopelovich, L., Abraham, S., McGrath, H., DeOme, K. B. and Chaikoff, I. L.—(1966) *Cancer Res.*, **26**, 1534.

Lewin, I., Bergel, F., Bray, R. C., Haddow, A. and Lewin, R.—(1957) *Proc. Am. Ass. Cancer Res.*, **2**, 226.

Lewin, I., Lewin, R. and Bray, R. C.—(1957) *Nature, Lond.*, **180**, 763.

Ling, E. R., Kon, S. K. and Porter, J. W. G.—(1961) In " Milk," edited by S. K. Kon and A. J. Cowie. New York (Academic Press), Vol. II.

Litwack, G., Bothwell, J. W., Williams, J. N. and Elvehjem, C. A.—(1953) *J. biol. Chem.*, **200**, 303.

Lowry, O. H., Rosebrough, N. J., Farr, A. L. and Randall, R. J.—(1951) *J. biol. Chem.*, **193**, 265.

Pitot, H. C.—(1963) *Cancer Res.*, **23**, 1474.

Ranadive, K. J. and Kanekar, S. A.—(1963) *Indian J. med. Res.*, **51**, 1005.

Reid, E. and Lewin, I.—(1957) *Br. J. Cancer*, **11**, 494.

Sheth, N. A., Wagle, M. M., Bhide, S. V. and Ranadive, K. J.—(1967) *Br. J. Cancer*, **21**, 228.

Westerfeld, W. W., Richert, D. A. and Hilfinger, M. F.—(1950) *Cancer Res.*, **10**, 486.